# Mask usage, social distancing, racial, and gender correlates of COVID-19 vaccine intentions among adults in the US

**DOI:** 10.1371/journal.pone.0246970

**Published:** 2021-02-16

**Authors:** Carl A. Latkin, Lauren Dayton, Grace Yi, Brian Colon, Xiangrong Kong

**Affiliations:** 1 Department of Health, Behavior and Society, Johns Hopkins Bloomberg School of Public Health, Johns Hopkins University, Baltimore, MD, United States of America; 2 Krieger School of Arts & Sciences, Johns Hopkins University, Baltimore, MD, United States of America; 3 Department of Ophthalmology, Johns Hopkins University School of Medicine, Johns Hopkins University, Baltimore, MD, United States of America; SUNY Downstate: SUNY Downstate Health Sciences University, UNITED STATES

## Abstract

Vaccine hesitancy could become a significant impediment to addressing the COVID-19 pandemic. The current study examined the prevalence of COVID-19 vaccine hesitancy and factors associated with vaccine intentions. A national panel survey by the National Opinion Research Center (NORC) was designed to be representative of the US household population. Sampled respondents were invited to complete the survey between May 14 and 18, 2020 in English or Spanish. 1,056 respondents completed the survey—942 via the web and 114 via telephone. The dependent variable was assessed by the item “If a vaccine against the coronavirus becomes available, do you plan to get vaccinated, or not?” Approximately half (53.6%) reported intending to be vaccinated, 16.7% did not intend, and 29.7% were unsure. In the adjusted stepwise multinominal logistic regression, Black and Hispanic respondents were significantly less likely to report intending to be vaccinated as were respondents who were females, younger, and those who were more politically conservative. Compared to those who reported positive vaccine intentions, respondents with negative vaccine intentions were significantly less likely to report that they engaged in the COVID-19 prevention behaviors of wearing masks (aOR = 0.53, CI = 0.37–0.76) and social distancing (aOR = 0.22, CI = 0.12–0.42). In a sub-analysis of reasons not to be vaccinated, significant race/ethnic differences were observed. This national survey indicated a modest level of COVID-19 vaccine intention. These data suggest that public health campaigns for vaccine uptake should assess in greater detail the vaccine concerns of Blacks, Hispanics, and women to tailor programs.

## Introduction

The death toll of COVID-19 cases and failed pandemic preparedness and response policies in the United States highlight the importance of an effective vaccine to halt the spread of SARS-CoV-2 (COVID-19) [1]. As of mid-December, 2020, the Pfizer- BioNTech vaccine (BNT162b2) had been approved in several countries, the Moderna vaccine (mRNA-1273) has a reported 94.5% efficacy, and the World Health Organization reported that many candidate vaccines were under clinical investigation [2]. Yet, vaccine hesitancy is likely to impair the effectiveness of the rollout of COVID-19 vaccine programs [3–5]. A May 2020 US national poll suggests that only about half of adults “plan to get vaccinated” if a vaccine against COVID-19 was accessible, and slightly less than a third reported that they were “not sure” if they would get vaccinated [6]. Given the low proportion of Americans who intend to be vaccinated, it is critical to examine factors associated with vaccine hesitancy so that programs can be developed to address these factors and encourage greater levels of COVID-19 vaccine acceptance and use. There are a wealth of studies on vaccine hesitancy, and prior research suggests that attitudes around vaccine hesitancy are difficult to change and are multifaceted, involving beliefs about individual freedoms, trust in government and pharmaceutical companies, and notions of health [7–12]. Further, it is well recognized that changing attitudes do not necessarily lead to changes in behaviors [13–15]. These findings suggest the importance of identifying other strategies to promote vaccine uptake.

Social identity theory argues that people identify with social categories that have normative behaviors. In turn, social identities both define and prescribe individuals’ attitudes and behaviors [16]. This theory suggests that encouraging people to engage in activities that promote an identity, especially a public identity consistent with COVID-19 prevention, could promote vaccine behaviors. Moreover, several theoretical perspectives (e.g., cognitive dissonance) suggest that engaging in behaviors inconsistent with attitudes may lead to attitude change [17]. It is therefore possible that engaging in COVID-19 prevention behaviors may impact COVID-19 vaccine attitudes, even among those with differing political attitudes. Consequently, in the current study, we examined whether the prevention behaviors of mask usage, social distancing, handwashing, and stocking up on food/supplies were associated with COVID-19 vaccine intentions as a positive association could both help guide the development of interventions to improve COVID-19 vaccine uptake as well as predict individuals and potentially geographic regions to target based on current levels of COVID-19 prevention behaviors.

In the current study, we used the National Opinion Research Center (NORC) US national survey data to examine whether the prevention behaviors, independently of political and demographic characteristics, were associated with vaccine intention [18]. These prevention behaviors differ in their private versus public nature, with handwashing and stocking up on food tending to be less public behavior than social distancing and mask wearing. The current analyses allowed us to also examine whether public COVID-19 prevention behaviors may be more strongly linked to vaccine intentions than more private behaviors. Additionally, as racial differences have been found regarding vaccine hesitancy and coverage, which may be partially due to medical mistrust, we also examine racial differences in vaccine hesitancy [19–21]. Identification of sub-groups who do not intend to be vaccinated or are hesitant to be vaccinated may help public health officials develop and target strategies for promoting COVID-19 vaccines when safe and effective vaccines become available.

## Materials and methods

The present study is based on publicly available survey data from the NORC Center for Public Affairs Research. Data were collected using the AmeriSpeak Omnibus®, which is a monthly survey of a probability-based panel designed to be representative of the US adult population. Randomly selected US households were sampled from the NORC National Sample Frame and then contacted by mail, email, telephone, and field interviewers. The panel is estimated to provide coverage for 97% of the US household population. Interviews for this survey were conducted with adults age 18 and older between May 14 and 18, 2020 residing in the 50 US states or the District of Columbia. This specific study sample was selected from the AmeriSpeak Panel using sampling strata based on age, race/ethnicity, education, and gender. Sampled AmeriSpeak respondents were invited to complete the survey through the member portal or a phone call from an interviewer. The panel reports a recruitment rate of 34%, with approximately 35,000 members. Panel members were randomly drawn from AmeriSpeak, and 1,056 completed the survey—942 via the web and 114 via telephone. Interviews were conducted in English and Spanish. The final stage completion rate was 12.7%, and the weighted household panel response rate was 24.1%. The research protocols were approved by the NORC and Johns Hopkins Bloomberg School of Public Health IRBs. The questionnaire and survey methodology are available at https://apnorc.org/download-data/. The data are available at https://apnorc.org/download-data/covid-19/ and the details of the AmeriSpeak methodology at https://amerispeak.norc.org/Documents/Research/AmeriSpeak%20Technical%20Overview%202019%2002%2018.pdf

### Measures

#### Vaccine intention

The dependent variable on vaccine intentions was assessed by the item, “If a vaccine against the coronavirus becomes available, do you plan to get vaccinated, or not?” The response options were “yes, I will get a coronavirus vaccine,” “no, I will not get a coronavirus vaccine,” and “not sure.”

#### COVID-19 social identity

Several “yes”/“no” questions assessed for COVID-19 social identity, defined as salient behaviors to address and prevent COVID-19. These questions used the stem, “Which of the following measures, if any, are you taking in response to the outbreak of the new coronavirus?” Answer options included “staying away from large groups,” “wearing a mask when leaving home,” “washing hands more frequently,” “stocking up on cleaning supplies,” and “stocking up on extra food.”

#### Covariates

To examine the saliency of COVID-19 in their lives, participants were asked, “Have you or has a close friend or relative been diagnosed with the coronavirus by a health care provider, or not (yes/no)?” Worrying about COVID-19 infection was assessed by asking, “How worried are you about you or someone in your family being infected with the coronavirus?” The response categories were on a 7-point scale from “extremely worried” to “not at all worried.” For the analyses, this variable was dichotomized at the median (extremely and very worried vs. somewhat, not too, and not at all worried) to represent higher and lower worry. Political ideology was assessed as a continuous variable from “very liberal” to “very conservative” using a 5-point scale. Respondents were also asked if they have “been unable to pay a credit card bill” because of the COVID-19 outbreak. The question, “How would you describe the community you live in now?” included the response options of “urban,” “rural,” and “suburban.” Age, race/ethnicity, gender, educational achievement, marital status, employment status, and income were also assessed.

#### Reasons for not getting a COVID-19 vaccine

Individuals who responded, “no, I will not get a coronavirus vaccine,” were also asked, “Which of the following are reasons you would not get a coronavirus vaccine?” Respondents could select multiple reasons and response options included “I am allergic to vaccines,” “I don’t like needles,” “I’m not concerned about getting seriously ill from the coronavirus,” “I won’t have time to get vaccinated,” “I would be concerned about getting infected with the coronavirus from the vaccine,” “I would be concerned about side effects from the vaccine,” “I don’t think vaccines work very well,” and “the coronavirus outbreak is not as serious as some people say it is.”

### Analyses

Of the 1,056 respondents, 1,043 provided data on vaccine intentions and hence were included in the analyses. We used bivariate and multivariate multinomial regression models to examine differences among respondents who reported that they did not intend to get the COVID-19 vaccine, when available, compared with those who reported intention to be vaccinated. The multinomial model also assessed the difference between those who were not sure if they would get vaccinated compared to those who intended to get vaccinated. Age and political conservatism were treated as ordinal variables. For regression models, education was dichotomized into high school education versus some college or more. Household income was dichotomized with a median split at $50,000. Multivariable models assessed the relationship between COVID-19 social identity and vaccine intentions (negative vs. positive and unsure vs. positive), adjusting for covariates. Two-sided Fisher’s Exact Test of independence was used to conduct the sub-analysis of racial/ethnic differences in reasons for not planning to be vaccinated among individuals who responded “no” to the vaccine intention question (N = 174).

In the first step of the multivariate multinomial regression models, all demographic variables, regardless of their statistically significant differences in bivariate associations, were included. In the second step, backward stepwise regression was used, with a criterion for retention of p < .10. We also modeled the data with forward stepwise regression and a model with all the variables included and found no appreciable differences in the models. A stepwise approach was used to develop a parsimonious model, as it was anticipated that several of the independent variables would be correlated, p<0.05 was considered statistically significant.

There was minimal missing data (Table 1). For three variables with missing data, linear imputation was used with rounding to the nearest integer to replace missing data for the multivariate regression analysis (missing data: community size, n = 10; worried about COVID-19 affecting family or self, n = 9; political ideology, n = 33). For the two other variables with missing data, the “don’t know” and “missing” responses were recoded as ‘no’ (missing data: unable to pay a credit card bill, n = 4; close friend or relative diagnosed with COVID-19, n = 4). In a sensitivity analysis conducted with models that excluded these cases, the observed magnitudes of association did not change by any appreciable amount.

**Table 1:** Descriptive statistics for COVID-19 vaccine intentions among NORC national sample (N = 1043).

	Total	Yes, I will get the coronavirus vaccine	No, I will not get the coronavirus vaccine	Not sure
	(N = 1043)	(n = 559; 53.60%)	(n = 174; 16.68%)	(n = 310; 29.72%)
	n (%)	n (%)	n (%)	n (%)
**Age**				
18 to 29	71 (6.8)	28 (5.0)	25 (14.4)	18 (5.8)
30 to 39	147 (14.1)	60 (10.7)	28 (16.1)	59 (19.0)
40 to 59	373 (35.8)	166 (29.7)	72 (41.4)	135 (43.6)
60 to 64	118 (11.3)	70 (12.5)	19 (10.9)	29 (9.4)
65 or older	334 (32.0)	235 (42.0)	30 (17.2)	69 (22.3)
**Ethnicity**				
White	724 (69.4)	437 (78.2)	102 (58.6)	185 (59.7)
Non-Hispanic Black	111 (10.6)	33 (5.9)	35 (20.1)	43 (13.9)
Hispanic	135 (12.9)	52 (9.3)	30 (17.2)	53 (17.1)
Other	73 (7.0)	37 (6.6)	7 (4.0)	29 (9.4)
**Gender** (Female)	731 (70.1)	355 (63.5)	127 (73.0)	249 (80.3)
**Education**				
High school and below	203 (19.5)	90 (16.1)	36 (20.7)	77 (24.8)
Some college and above	840 (80.5)	469 (83.9)	138 (79.3)	233 (75.2)
**Marital Status** (Married)	627 (60.1)	337 (60.3)	97 (55.8)	193 (62.3)
**Employment Status**				
Employed	591 (56.7)	298 (53.3)	108 (62.1)	185 (59.7)
**Income**				
Less than $50,000	447 (42.9)	207 (37.0)	96 (55.2)	144 (46.5)
$50,000 or more	596 (57.1)	352 (63.0)	78 (44.8)	166 (53.5)
**Community type**^**1**^				
Urban	297 (28.8)	165 (29.7)	47 (27.3)	85 (27.8)
Suburban	481 (46.6)	270 (48.6)	77 (44.8)	134 (43.8)
Rural	255 (24.7)	120 (21.6)	48 (27.9)	87 (28.4)
**Worried about COVID-19 infecting family or self** ^**2**^				
Extremely Worried	215 (20.8)	129 (23.3)	37 (21.5)	49 (15.9)
Very Worried	225 (21.8)	135 (24.4)	17 (9.9)	73 (23.7)
Somewhat Worried	341 (33.0)	200 (36.1)	38 (22.1)	103 (33.4)
Not too Worried	179 (17.3)	76 (13.7)	42 (24.4)	61 (19.8)
Not at all Worried	74 (7.2)	14 (2.5)	38 (22.1)	22 (7.1)
**Self or others diagnosed with COVID-19** (Yes)	192 (18.4)	93 (16.6)	43 (24.7)	56 (18.1)
**Behaviors taken to prevent COVID** (Yes)				
Staying away from large groups	947 (90.8)	538 (96.2)	130 (74.7)	279 (90.0)
Wearing masks	825 (79.1)	486 (86.9)	98 (56.3)	241 (77.7)
Frequent hand washing	962 (92.2)	532 (95.2)	145 (83.3)	285 (91.9)
Stocking up on supplies	391 (37.5)	213 (38.1)	66 (37.9)	112 (36.1)
Stocking up on food	441 (42.3)	253 (45.3)	71 (40.8)	117 (37.7)
**Unable to make credit card payment due to COVID**	129 (12.4)	51 (9.1)	34 (19.5)	44 (14.2)
**Political orientation**^**3**^	** **			
Very Liberal	96 (9.5)	69 (12.6)	13 (7.8)	14 (4.7)
Somewhat Liberal	145 (14.4)	100 (18.2)	13 (7.8)	32 (10.8)
Moderate	486 (48.1)	271 (49.5)	63 (37.7)	152 (51.5)
Somewhat Conservative	169 (16.7)	65 (11.9)	41 (24.6)	63 (21.4)
Very Conservative	114 (11.3)	43 (7.8)	37 (22.2)	34 (11.5)

^1^ These values reflect a response count of N = 1033.

^2^ These values reflect a response count of N = 1034.

^3^ These values reflect a response count of N = 1010.

## Results

In the study sample, most were in the age groups of 40–59 (35.8%) or 65 or older (32.0%). Most (56.7%) were employed, White (69.4%), and almost half (46.6%) lived in suburban areas (Table 1). In both bivariate multinomial regression models (Table 2), COVID-19 vaccine intention was significantly associated with sociodemographic variables including race (Black, Hispanic, Mixed/other, and White), age group, binary gender, employment status, educational attainment, income level, and political ideology. Participants’ concern that they or someone in their family would become infected was significantly associated with the COVID-19 vaccine intentions in both models. Having already been diagnosed or having a close friend or relative diagnosed with COVID-19 by a healthcare provider was not significantly associated with vaccine intention. Social distancing and mask usage were significantly associated with vaccine intention in the two models, and handwashing was significant in one model (yes vs. no).

**Table 2:** Unadjusted and adjusted multinomial logistic regression models of COVID-19 vaccine intention.

	Model 1		Model 2	
	No		Not Sure	
	(Ref: Yes)		(Ref: Yes)	
	OR	aOR	OR	aOR
	(95% CI)	(95% CI)	(95% CI)	(95% CI)
**Age group** (continuous)*	**0.61**	**0.68**	**0.71**	**0.78**
	**(0.53–0.70)**	**(0.57–0.82)**	**(0.63–0.79)**	**(0.67–0.90)**
Ethnicity* (Ref: White)	**REF**	**REF**	**REF**	**REF**
Non-Hispanic Black	**4.54**	**6.34**	**3.08**	**3.47**
	**(2.70–7.66)**	**(3.46–11.60)**	**(1.90–5.00)**	**(2.04–5.88)**
Hispanic	**2.47**	**2.27**	**2.41**	**2.1**
	**(1.50–4.07)**	**(1.26–4.08)**	**(1.58–3.66)**	**(1.31–3.39)**
Mixed/other	0.81	1.03	**1.85**	**2.13**
	(0.35–1.87)	(0.41–2.55)	**(1.11–3.10)**	**(1.22–3.71)**
Marital Status* (Ref: Married)	0.83	1.02	1.09	1.25
	(0.59–1.17)	(0.66–1.57)	(0.82–1.45)	(0.89–1.76)
Gender* (Ref: Female)	**1.55**	**1.74**	**2.35**	**2.49**
	**(1.07–2.26)**	**(1.12–2.71)**	**(1.67–3.26)**	**(1.73–3.58)**
Employment Status* (Ref: Employed)	**0.7**	1.01	0.77	0.96
	**(0.49–0.99)**	(0.65–1.57)	(0.58–1.02)	(0.68–1.35)
Income Level*	**0.48**	**0.52**	**0.68**	0.78
(Ref: Less than $50,000)	**(0.34–0.68)**	**(0.33–0.82)**	**(0.51–0.90)**	(0.55–1.12)
Education Level*	0.74	1.27	**0.58**	0.73
(Ref: High school and below)	(0.48–1.13)	(0.76–2.13)	**(0.41–0.82)**	(0.50–1.08)
**Level of worry about COVID-19^** (Ref: Not at all worried)	**0.5**	0.66	**0.72**	**0.69**
	**(0.35–0.72)**	(0.43–1.03)	**(0.54–0.95)**	**(0.50–0.95)**
**Political Orientation^** (Continuous)	**1.74**	**1.82**	**1.46**	**1.54**
	**(1.47–2.07)**	**(1.49–2.23)**	**(1.28–1.68)**	**(1.32–1.79)**
**Staying away from groups^,^$^**	**0.12**	**0.22**	**0.35**	**0.49**
	**(0.07–0.20)**	**(0.12–0.42)**	**(0.20–0.62)**	**(0.26–0.92)**
**Wearing a mask^**	**0.19**	**0.34**	**0.53**	0.69
	**(0.13–0.29)**	**(0.21–0.54)**	**(0.37–0.76)**	(0.46–1.05)
**Community Type^** (Ref: Rural)	**REF**	**REF**	**REF**	**REF**
Urban	0.71	---	0.72	---
	(0.45–1.13)		(0.50–1.06)	
Suburban	0.71	---	**0.69**	---
	(0.47–1.08)		**(0.49–0.98)**	
**Frequent Hand Washing^, ^$^**	**0.25**	---	0.58	---
	**(0.15–0.44)**		(0.33–1.02)	
**Stocking up on supplies^, ^$^**	0.99	---	0.92	---
	(0.70–1.41)		(0.69–1.23)	
**Stocking up on food^, ^$^**	0.83	---	**0.73**	---
	(0.59–1.18)		**(0.55–0.97)**	
**Unable to pay Credit Card^, ^$^**	**0.41**	---	**0.61**	---
	**(0.26–0.66)**		**(0.40–0.93)**	
**Self or others diagnosed with COVID-19^,^$^**	**0.61**	---	0.91	---
	**(0.40–0.92)**		(0.63–1.30)	

Note

*-Variables forced entered into the model

^-Variable backward stepwise entry. Missing aOR values indicate variables that were not included in the final model

$-Dichotomous, “No” was the reference group.

In both of the backward stepwise regression model, several covariates were removed from the final, parsimonious model: community type, frequent hand-washing, stocking up on cleaning supplies or extra food, inability to pay a credit card bill, and COVID-19 diagnosis for self, family, or friends.

As shown in the final model comparing those with negative vaccine intention to positive intention (Table 2), several sociodemographic variables were independently related to the negative intention to obtain a COVID-19 vaccine. Compared to White participants, Black (aOR = 6.34 95% CI = 3.46–11.60) and Hispanic (aOR = 2.27, 95% CI = 1.26–4.08) respondents were significantly more likely to report that they did not intend to obtain a COVID-19 vaccine, if available. Gender was also independently related to vaccine intention, with women being more likely than men to report negative COVID-19 vaccine intention (aOR = 1.74, 95% CI = 1.12–2.71). The increasing age of respondents was also associated with reduced reports of negative vaccine intention (aOR = 0.68, 95% CI = 0.57–0.82). Respondents with an annual income above $50,000 were also less likely to report that they would not obtain a COVID-19 vaccine, if available (aOR = 0.52, 95% CI = 0.33–0.82). Political ideology was independently related to vaccine intention, with increasingly conservative ideology significantly associated with not intending to obtain a potential COVID-19 vaccine (aOR = 1.82, 95% CI = 1.49–2.23).

The item of how worried respondents were that they or a family member would become infected was not significantly associated with intending to obtain a COVID-19 vaccine if available. Interestingly, only the more public preventive behaviors–i.e., social distancing and mask usage–were associated with positive vaccine intention. Those who reported negative vaccine intentions, compared to the positive intention group, had reduced odds of more frequent social distancing and mask usage (aOR = 0.22, 95% CI = 0.12–0.42 and aOR = 0.34, 95% CI = 0.21–0.54, respectively).

The results of the component of the multinomial regression Model 2, which compared those reporting uncertain (“not sure”) intention of obtaining a COVID-19 vaccine to those with positive vaccine intentions (Table 2) identified several sociodemographic factors significantly associated with unsure intention. Compared to White participants, Black participants were more likely to report that they were “not sure” about their intention to obtain a vaccine compared to reporting a positive vaccine intention (aOR = 3.47, 95% CI = 2.04–5.88). Female gender and political conservatism were associated with higher odds of reporting uncertain vaccine intention compared to positive vaccine intention (aOR = 2.49, 95% CI = 1.73–3.58 and aOR = 1.54, 95% CI = 1.32–1.79, respectively). Level of worry about COVID-19 infection was associated with reduced odds of being in the uncertain vaccine intention compared to the positive intention group (aOR = 0.69, 95% CI = 0.50–0.95). Similar to results from multivariate Model 1, more public COVID-19 preventive behaviors were significantly associated with reduced uncertainty about obtaining a vaccine. Staying away from large groups (social distancing) was associated with being less likely to report vaccine uncertainty compared to positive vaccine intentions (aOR = 0.49, 95% CI = 0.26–0.92). Similarly, in bivariate models, mask-wearing was associated with reduced odds of being in the uncertain vaccine intentions group compared to the positive vaccine intentions group (OR = 0.53, 95% CI = 0.37–0.76); however, this finding did not remain significant in the multivariable model.

In a sub-analysis, we analyzed racial/ethnic differences in vaccine hesitancy among individuals who responded “no” to the vaccine intention question (N = 174, Table 3). Fisher’s Exact Tests indicated significant racial/ethnic differences among participants who reported that they would not obtain a vaccine due to a lack of time to get vaccinated (p = 0.013, Fisher’s exact test). Descriptive analyses showed that a proportionately greater number of Black (5.7%) and Hispanic respondents (10.0%) reported a lack of time to get vaccinated as a reason for vaccine refusal compared with 0% of White or “Other race/ethnicity” participants. Fisher’s Exact Test results also revealed racial/ethnic differences in vaccine refusal due to concern about getting infected with COVID-19 from the vaccine (p = 0.004, Fisher’s exact test). Approximately 66% of Black participants cited this concern, followed by 47% Hispanic, 34% White, and 14% “Other race/ethnicity.” Results further indicated that there were significant racial/ethnic differences among respondents who reported that they would not obtain a COVID-19 vaccine if available because the COVID-19 outbreak “is not as serious as some people say it is,” (p = 0.037, Fisher’s exact test). A proportionately greater number of White participants (31.4%) endorsed this reason, as compared with 14.3% of Black and “Other race/ethnicity” participants and 10.0% of Hispanic participants. There were no significant differences in race/ethnicity among respondents who reported vaccine hesitancy/refusal due to allergy to vaccines, dislike of needles, lack of concern about getting seriously ill from COVID-19, concern about vaccine side effects, or belief in vaccine efficacy.

**Table 3:** Racial/ethnic differences in reported reasons for not intending to obtain a COVID-19 vaccine (N = 174).

Which of the following are reasons you would not get a coronavirus vaccine?	African-American	Hispanic	Other	White, non-Hispanic	Fisher’s Exact Test (two-sided)
	(n = 35, 20.1%)	(n = 30; 17.2%)	(n = 7; 4.10%)	(n = 102; 58.60%)	p-value
I am allergic to vaccines	3 (8.6)	0 (0.0)	0 (0.0)	7 (6.9)	0.454
I don’t like needles	6 (17.1)	6 (20.0)	0 (0.0)	9 (8.8)	0.218
I’m not concerned about getting seriously ill from the coronavirus	10 (28.6)	4 (13.3)	2 (28.6)	37 (36.3)	0.099
I won’t have time to get vaccinated	2 (5.7)	3 (10.0)	0 (0.0)	0 (0.0)	**0.013**
I would be concerned about getting infected with the coronavirus from the vaccine	23 (65.7)	14 (46.7)	1 (14.3)	35 (34.3)	**0.004**
I would be concerned about side effects from the vaccine	26 (74.3)	19 (63.3)	6 (85.7)	73 (71.6)	0.661
I don’t think vaccines work very well	13 (37.1)	6 (20.0)	3 (42.9)	33 (32.4)	0.404
The coronavirus outbreak is not as serious as some people say it is	5 (14.3)	3 (10.0)	1 (14.3)	32 (31.4)	**0.037**

## Discussion

From this well characterized US national representative study, we found that the three groups of vaccine intention (yes/no/not sure) significantly differed based on background factors and COVID-19 social identity. Not surprisingly, those with positive COVID-19 vaccine intention were more different from those with negative vaccine intention than those with unsure intention. Black and Hispanic respondents were significantly less likely to report intending to obtain a vaccine than White respondents. Surprisingly, women were less likely to report intending to be vaccinated, which differs from results indicated by prior studies [22]. These results suggest that a COVID-19 social identity, as assessed through engagement in COVID-19 prevention behaviors, is associated with vaccine intention. With regard to COVID-19 preventive measures, the more public behaviors of mask usage and social distancing were strongly associated with vaccine intentions, whereas handwashing and stocking up on food/supplies were not associated in the multivariate models. This finding is consistent with social identity theory, as handwashing and stocking up on supplies are more private preventive behaviors and thus may differ from the more public COVID-19 prevention behaviors. Additionally, handwashing and stocking of food/supplies may not be strong indicators of COVID-19-prevention identity as they are not behaviors specific to COVID-19 prevention. We do not know if there is a causal association between the COVID-19 prevention identity and vaccine intention variables, and hence this warrants a longitudinal assessment. Political conservativism was found to be associated with not intending to be vaccinated. Prior research on vaccine hesitancy has found mixed results on the role of political ideology on vaccine hesitancy [23, 24]. We do not know if the current politicization and polarization of COVID-19 have had a unique impact on COVID-19 vaccine hesitancy.

This study is subject to several limitations. It is unknown whether hesitancy predicts actual behaviors or if COVID-19 vaccine hesitancy will change when a vaccine is developed, either based on the vaccine’s effectiveness/side-effects, or as a result of the future political climate. The study is also limited by the cross-sectional design as well as its reliance on self-reported data. Consequently, we do not know if a heightened identity of COVID-19 prevention or engaging in COVID-19 prevention behaviors will lead to greater vaccine acceptance. Moreover, since it was designed to be nationally representative, high risk and some minority subgroups had small cell sizes, limiting analyses and inferences. Another limitation is that the study focused only on individual-level factors rather than structural barriers to vaccine uptake. It may be more fruitful to also conceptualize vaccine uptake as a community and systems-level variable and provide community-level incentives for high vaccination rates. In addition, there were no criteria provided to respondents in selection of community type (rural, suburban, urban). Participants’ self-identification of community type may vary in accuracy and limit the interpretability of particular study findings relevant to community size. Furthermore, study findings may be less representative of certain populations whom, for example, may have high levels of distrust for the media and polls and hence did not respond. Moreover, social desirability bias is not likely to be randomly distributed and may have impacted the study findings as well as the mode of survey administration.

The lower likelihood of Black and Hispanic participants to report intending to obtain a COVID-19 vaccine when available is disconcerting, especially given the COVID-19 mortality disparities with much higher rates among Black and Hispanic patients in the US [25, 26]. A sub-analysis among respondents who did not plan to obtain a COVID-19 vaccine indicated that Blacks, compared to Whites, were almost twice as likely to report concerns about becoming infected from the vaccine. In contrast, Whites, compared to other racial/ethnic groups, were more than twice as likely to report that one of the reasons for not intending to get a vaccine was that “the coronavirus outbreak is not as serious as some people say it is.” These findings are from a subsample and highlight the importance of studies examining racial/ethnic differences in vaccine intentions.

These data suggest that public health campaigns for vaccine uptake should assess in greater detail the vaccine concerns of Black and Hispanic US residents to tailor vaccine uptake programs. Future research should longitudinally examine whether social distancing and mask usage policies enacted by states, counties, and cities have an impact on vaccine hesitancy as well as monitor vaccine hesitancy in real-time to help predict levels of vaccine uptake and inform future public health campaigns aiming to improve vaccination rates. A high degree of hope has been placed on a vaccine to eradicate SARS-CoV-2. However, unless there are active and targeted campaigns to foster vaccine uptake and access, the public health impact of an effective vaccine is uncertain.
