# Supplementary material for: Mask usage, social distancing, racial, and gender correlates of COVID-19 vaccine intentions among adults in the US
Source: PLoS One. 2021 Feb 16;16(2):e0246970. doi: 10.1371/journal.pone.0246970 (PMC7886161; doi:10.1371/journal.pone.0246970)
Supplement: Attachment — Submitted filename: Response to Reviewers.docx [file pone.0246970.s001.docx]

**Dear Editors,**

**Below please find in bold the responses to the editors’ and reviewers’ questions.**

Journal Requirements:

When submitting your revision, we need you to address these additional requirements

1. Please ensure that your manuscript meets PLOS ONE's style requirements, including those for file naming. The PLOS ONE style templates can be found at

[https://journals.plos.org/plosone/s/file?id=wjVg/PLOSOne_formatting_sample_main_body.pdf](https://nam02.safelinks.protection.outlook.com/?url=https%3A%2F%2Fjournals.plos.org%2Fplosone%2Fs%2Ffile%3Fid%3DwjVg%2FPLOSOne_formatting_sample_main_body.pdf&data=04%7C01%7Cldayton2%40jhu.edu%7Cb03b07dde6154aa2568f08d89c5fce39%7C9fa4f438b1e6473b803f86f8aedf0dec%7C0%7C0%7C637431282578178073%7CUnknown%7CTWFpbGZsb3d8eyJWIjoiMC4wLjAwMDAiLCJQIjoiV2luMzIiLCJBTiI6Ik1haWwiLCJXVCI6Mn0%3D%7C1000&sdata=X1svT67ZuAcit%2FHOw85ykhw0uQLzTQ8DOH%2Blnb5gSfY%3D&reserved=0) and

[https://journals.plos.org/plosone/s/file?id=ba62/PLOSOne_formatting_sample_title_authors_affiliations.pdf](https://nam02.safelinks.protection.outlook.com/?url=https%3A%2F%2Fjournals.plos.org%2Fplosone%2Fs%2Ffile%3Fid%3Dba62%2FPLOSOne_formatting_sample_title_authors_affiliations.pdf&data=04%7C01%7Cldayton2%40jhu.edu%7Cb03b07dde6154aa2568f08d89c5fce39%7C9fa4f438b1e6473b803f86f8aedf0dec%7C0%7C0%7C637431282578178073%7CUnknown%7CTWFpbGZsb3d8eyJWIjoiMC4wLjAwMDAiLCJQIjoiV2luMzIiLCJBTiI6Ik1haWwiLCJXVCI6Mn0%3D%7C1000&sdata=9paYQG8ee0dLS0u33cf1NWLVr6sHZ0pbAk%2FtAtahnzo%3D&reserved=0)

**We have now used the PLOS ONE's style requirements**

2. Please include additional information regarding the survey or questionnaire used in the study and ensure that you have provided sufficient details that others could replicate the analyses. For instance, if you developed a questionnaire as part of this study and it is not under a copyright more restrictive than CC-BY, please include a copy, in both the original language and English, as Supporting Information, or include a citation if it has been published previously.

**We have provided the web link to the survey and data.**

3. In the Methods, please discuss whether and how the questionnaire was validated and/or pre-tested.

**We now include a web link to the larger study's methods and methodologies that provide copious details on the study methods. The survey items on vaccine intentions were based on items on seasonal influenzas vaccine intentions from CDC.**

4. PLOS requires an ORCID iD for the corresponding author in Editorial Manager on papers submitted after December 6th, 2016. Please ensure that you have an ORCID iD and that it is validated in Editorial Manager. To do this, go to 'Update my Information' (in the upper left-hand corner of the main menu), and click on the Fetch/Validate link next to the ORCID field. This will take you to the ORCID site and allow you to create a new iD or authenticate a pre-existing iD in Editorial Manager. Please see the following video for instructions on linking an ORCID iD to your Editorial Manager account: [https://www.youtube.com/watch?v=_xcclfuvtxQ](https://nam02.safelinks.protection.outlook.com/?url=https%3A%2F%2Fwww.youtube.com%2Fwatch%3Fv%3D_xcclfuvtxQ&data=04%7C01%7Cldayton2%40jhu.edu%7Cb03b07dde6154aa2568f08d89c5fce39%7C9fa4f438b1e6473b803f86f8aedf0dec%7C0%7C0%7C637431282578188070%7CUnknown%7CTWFpbGZsb3d8eyJWIjoiMC4wLjAwMDAiLCJQIjoiV2luMzIiLCJBTiI6Ik1haWwiLCJXVCI6Mn0%3D%7C1000&sdata=%2F8ikkQL2lH4TA3KuO%2B%2BcOOzST9fiFFekPiQ3Sqf0fI0%3D&reserved=0)

**We include the ORCID iD for the corresponding author (0000-0002-7931-2116)**

5. We note you have included a table to which you do not refer in the text of your manuscript. Please ensure that you refer to Table 3 in your text; if accepted, production will need this reference to link the reader to the Table.

**We have now included the reference for Table 3 in the text.**

Reviewers' comments:

Reviewer's Responses to Questions

**Comments to the Author**

1. Is the manuscript technically sound, and do the data support the conclusions?

The manuscript must describe a technically sound piece of scientific research with data that supports the conclusions. Experiments must have been conducted rigorously, with appropriate controls, replication, and sample sizes. The conclusions must be drawn appropriately based on the data presented.

Reviewer #1: Partly

2. Has the statistical analysis been performed appropriately and rigorously?

Reviewer #1: Yes

3. Have the authors made all data underlying the findings in their manuscript fully available?

The [PLOS Data policy](https://nam02.safelinks.protection.outlook.com/?url=http%3A%2F%2Fwww.plosone.org%2Fstatic%2Fpolicies.action%23sharing&data=04%7C01%7Cldayton2%40jhu.edu%7Cb03b07dde6154aa2568f08d89c5fce39%7C9fa4f438b1e6473b803f86f8aedf0dec%7C0%7C0%7C637431282578188070%7CUnknown%7CTWFpbGZsb3d8eyJWIjoiMC4wLjAwMDAiLCJQIjoiV2luMzIiLCJBTiI6Ik1haWwiLCJXVCI6Mn0%3D%7C1000&sdata=aDak3YNoDckq1khZvQ%2FFiufWCs6F8j7Prp5w%2BQqvFGw%3D&reserved=0) requires authors to make all data underlying the findings described in their manuscript fully available without restriction, with rare exception (please refer to the Data Availability Statement in the manuscript PDF file). The data should be provided as part of the manuscript or its supporting information, or deposited to a public repository. For example, in addition to summary statistics, the data points behind means, medians and variance measures should be available. If there are restrictions on publicly sharing data—e.g. participant privacy or use of data from a third party—those must be specified.

Reviewer #1: Yes

4. Is the manuscript presented in an intelligible fashion and written in standard English?

PLOS ONE does not copyedit accepted manuscripts, so the language in submitted articles must be clear, correct, and unambiguous. Any typographical or grammatical errors should be corrected at revision, so please note any specific errors here.

Reviewer #1: Yes

5. Review Comments to the Author

Please use the space provided to explain your answers to the questions above. You may also include additional comments for the author, including concerns about dual publication, research ethics, or publication ethics. (Please upload your review as an attachment if it exceeds 20,000 characters)

Reviewer #1: Very interesting and informative study that could help guide messages to improve vaccine adherence.

A couple of points to address:

Intro
- The first sentence of the last paragraph (In the current study...) - appears to be an incomplete sentences

**We have edited this sentence for clarity.**

Methods
- What were the criteria (or examples given to respondents, if any) for urban, suburban or rural communities respondents live in?

**Community size was assessed only by the question: “How would you describe the community you live in now?” Respondents were provided the options of “urban,” “rural,” and “suburban” and self-reported their community size. Of the sample, 24.8% were rural, 28.5% urban, and 6.5% suburban, which suggests a geographically diverse sample. We have referred the readers to 2 detailed appendices on the recruitment and enrollment, which also provides the descriptive statistics, on all the variables, as well as included additional text about the criteria.**

**The only enrollment criteria included adults age 18 and older who were residing in the 50 states and the District of Columbia at the time of survey. As the goal of the panel was to be representative of the US adult population, albeit with weighting, it was open to all adults.  In addition to developing national representative sample, the panel was designed to be representative to eight US regions.**

Results
- There is a discrepancy in how the the variables are treated in Table 1 and Table 2. In Table 1, the variable age for example, is treated as categorical but linear in Table 2. The same for Level of Worry. If they are intended to treated as categorical in table 2, please adjust tables and ensure all variables have identified references with justifications.

**Tables 1 and 2 now consistently reflect the categorical ‘Level of Worry’ variable and Table 2 provides the appropriate reference category. We also clarified that we used the same variables in the two tables. In addition, we have now identified the reference categories for all the categorical variables in Table 2 to clarify that they were treated the same in both tables**

Discussion
- If there were no criteria for respondents selection of community type, please address in limitations

**We now include in the limitation section that there were no criteria for respondents’ self-report of community type. The following information was added to the Limitations section:**

**“In addition, there were no criteria provided to respondents in selection of community type (rural, suburban, urban). Participants’ self-identification of community type may be the sample may be inaccurate and limit the interpretability of particular study findings relevant to community size.”**

**We also include the limitation that the sample is less representative of certain populations** **whom, for example, may have high levels distrust for the media and polls and hence did not respond. Moreover, social desirability bias is not likely to be randomly distributed and may have had an impact on the study findings.**
- Given abundance of misinformation found on the internet, would recommend discussing the proportion of web-based respondents as a potential limitation

**We now state that we also do not know how the mode of administration may be linked to response biases in this survey. The following has been added to the “Limitations” section:**

**“Furthermore, study findings may be less representative of certain populations whom, for example, may have high levels of distrust for the media and polls and hence did not respond. Moreover, social desirability bias is not likely to be randomly distributed and may have impacted the study findings as well as the mode of survey administration.”**
